# LncRNA HOTAIR: A Potential Prognostic Factor and Therapeutic Target in Human Cancers

**DOI:** 10.3389/fonc.2021.679244

**Published:** 2021-07-22

**Authors:** Xiaoru Xin, Qianan Li, Jinyong Fang, Tiejun Zhao

**Affiliations:** ^1^College of Chemistry and Life Sciences, Zhejiang Normal University, Jinhua, China; ^2^Department of Science and Education, Jinhua Guangfu Oncology Hospital, Jinhua, China

**Keywords:** HOTAIR, cancer, prognosis, therapy, potential, drug resistance, survival, knockdown

## Abstract

Long non-coding RNAs (lncRNAs) are emerging as crucial regulators of gene expression and physiological processes. LncRNAs are a class of ncRNAs of 200 nucleotides in length. HOX transcript antisense RNA (HOTAIR), a trans-acting lncRNA with regulatory function on transcription, can repress gene expression by recruiting chromatin modifiers. HOTAIR is an oncogenic lncRNA, and numerous studies have determined that HOTAIR is highly upregulated in a wide variety of human cancers. In this review, we briefly summarize the impact of lncRNA HOTAIR expression and functions on different human solid tumors, and emphasize the potential of HOTAIR on tumor prognosis and therapy. Here, we review the recent studies that highlight the prognostic potential of HOTAIR in drug resistance and survival, and the progress of therapies developed to target HOTAIR to date. Furthermore, targeting HOTAIR results in the suppression of HOTAIR expression or function. Thus, HOTAIR knockdown exhibits great therapeutic potential in various cancers, indicating that targeting lncRNA HOTAIR may serve as a promising strategy for cancer therapy. We also propose that preclinical studies involving HOTAIR are required to provide a better understanding of the exact molecular mechanisms underlying the dysregulation of its expression and function in different human cancers and to explore effective methods of targeting HOTAIR and engineering efficient and targeted drug delivery methods *in vivo*.

## Introduction

The ENCODE project revealed that the majority of the human genome is actively transcribed, but only a small minority of the genome encode proteins (1). Transcribed RNAs that do not encode proteins are known as non-coding RNAs (ncRNAs), which include a subgroup of ncRNAs classified as long ncRNAs (lncRNAs) based on their length of >200 nucleotides (2, 3). Many identified lncRNAs are transcribed by RNA polymerase II (RNA pol II) from different regions in the genome (4–6). Based on the genomic location, lncRNAs are mainly classified into four groups: intergenic lncRNAs, intronic lncRNAs, overlapping lncRNAs, and antisense lncRNAs. A comprehensive classification of lncRNAs can be obtained from the review by Jarroux et al. (7). Most lncRNAs can regulate gene expression regardless of the subtype of lncRNAs.

Accumulating evidence has shown that lncRNAs play a critical role both in physiological processes and in human disease development including cancer. LncRNAs are known as key epigenetic regulators for gene expression (8) and are involved in various processes of cellular homeostasis, including chromatin modification, chromatin silencing, transcriptional regulation for gene, and transcription/functional regulation of microRNAs (miRNAs). Several regulatory capacities of lncRNAs are achieved given the relatively complex structure of lncRNAs, which endow the lncRNAs with the ability to bind to DNA partners, protein, and RNA (9). Thereby, the aberrant expression of lncRNAs, especially lncRNA-mediated dysregulation of normal physiological process, may lead to human diseases including cancer (10). In reality, abnormal expression and function of lncRNAs in human cancers has been widely reported, highlighting their capacity to influence oncogenesis, metastatic progression, recurrence, prognosis, and therapeutic responses (11).

**Figure 1:**
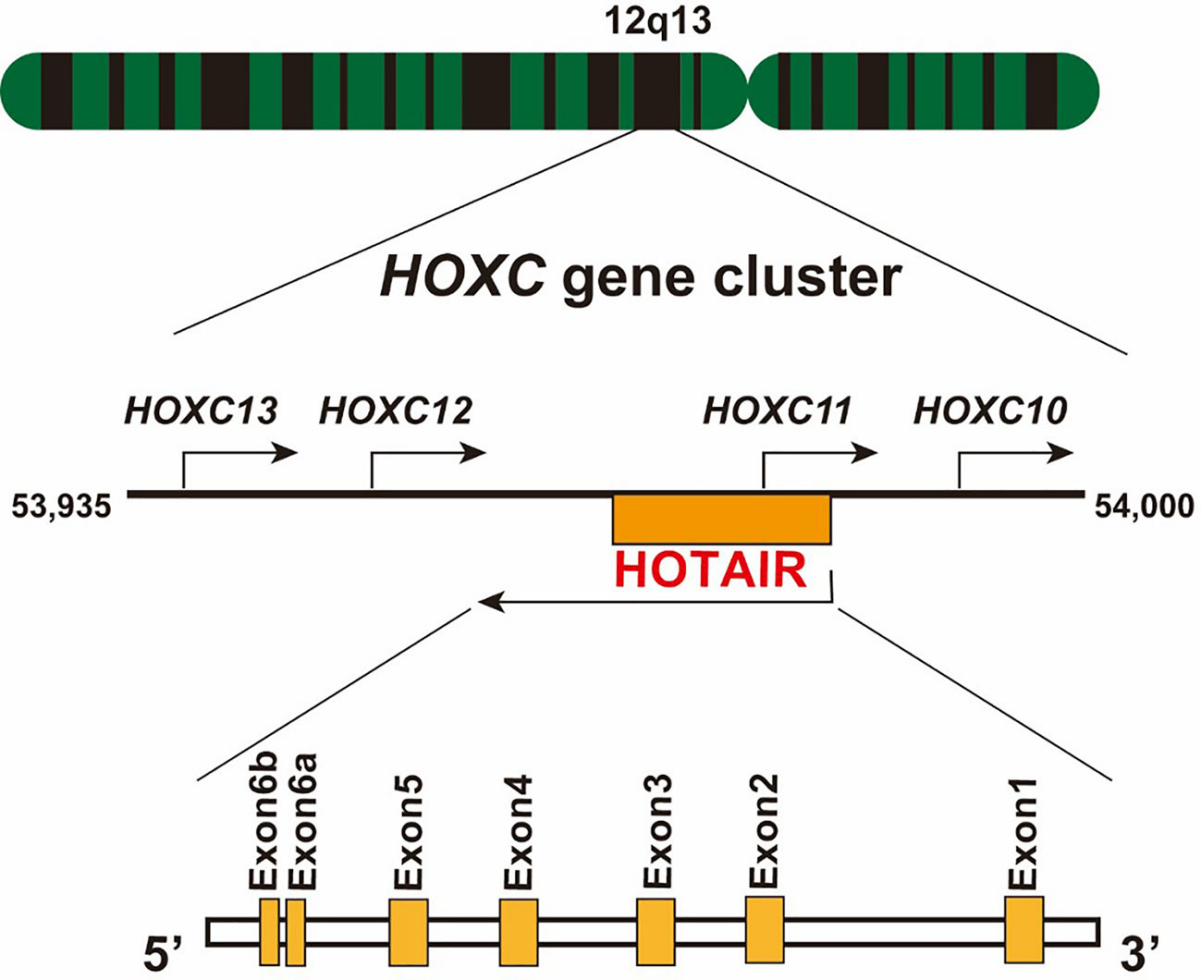
Schematic location of lncRNA HOTAIR. The HOTAIR gene is located in the center of the *HOXC* gene cluster on chromosome 12, specifically between *HOXC11* and *HOXC12*, on the antisense strand. It consists of six exons, and exon 6 contains two domains.

HOX transcript antisense RNA (*HOTAIR*), a trans-acting intergenic lncRNA, was first introduced by Rinn et al. as a polyadenylated and spliced RNA of 2,158 nucleotides in length (12, 13). In humans, HOTAIR is located on chromosome 12q13.13, between the *HOXC11* and *HOXC12* gene, and is transcribed in an antisense manner relative to the canonical *HOXC* genes, and partly overlapping with *HOXC11* (**Figure 1**). Human HOTAIR is composed of seven exons, with the last two exons being nearly adjacent to each other; therefore, they are defined as two domains of exon 6 (14). HOTAIR can form a complex secondary structure, comprising several stem and loop structures (15). Evolutionarily, HOTAIR is highly conserved and has evolved faster than its neighboring *HOXC* gene (16).

**Figure 2:**
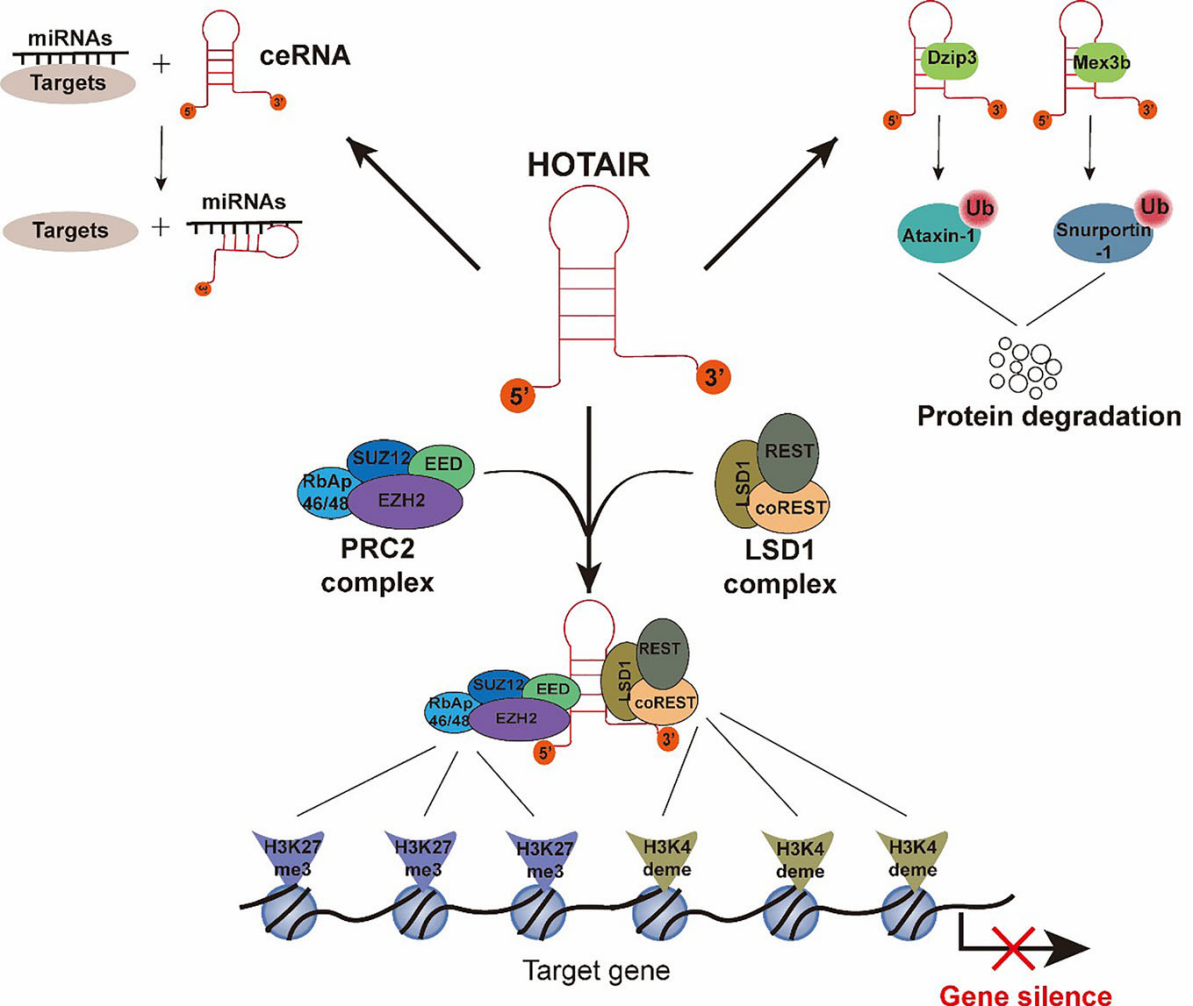
Functions and molecular mechanisms of HOTAIR. The interactions between HOTAIR and its several important partners are summarized. (1) The 5′-end of HOTAIR binds to the PRC2 complex. The 3′-end of HOTAIR binds to the LSD1 complex. H3K27 tri-methylation and H3K4me3 demethylation result from PRC2 complex and LSD1 complex activity, respectively, and cause gene silencing. (2) HOTAIR interacts with E3 ubiquitin ligases, Dzip3 and Mex3b, and facilitates the ubiquitination of Ataxin-1 and Snurportin-1, thereby contributing to their degradation. (3) HOTAIR interacts with miRNAs as a competitive endogenous RNA to promote the expression of miRNA-targeted genes.

HOTAIR is a crucial regulator of chromatin status and gene transcriptional silencing (17). Previous studies have largely determined that the mechanisms of HOTAIR, serving as an important epigenetic regulator, depends on interactions with protein or RNA partners. To date, four main molecules indispensable for HOTAIR’s function have been studied (18) (**Figure 2**). The most widely described partner is Polycomb repressive complex 2 (PRC2). PRC2 is a protein complex, which can mark a gene for transcriptional repression through tri-methylation of histone H3 Lys 27 (H3K27me3) (19, 20). The PRC2 complex contains four major subunits, namely, EZH2, EED, SUZ12, and RbAp46/48 (21). Although EZH2 is the critical subunit for the methyltransferase process, three other subunits are also essential for the EZH2 catalytic activity (22). Early studies have shown that HOTAIR is capable of binding to the PRC2 with an 89 bp fragment on the 5’ end (**Figure 2**) **(**12, 23, 24), and HOTAIR is necessary for PRC2 occupancy and H3K27me3 formation in different chromosomes (17). HOTAIR can bind a DNA polypurine motif to regulate gene transcription (25). Other researchers have proposed a different mechanism for the interaction between PRC2 and HOTAIR, in which the PRC2 complex interacts with HOTAIR through the short repeats of the consecutive guanines in the HOTAIR sequence, rather than with a specific structural domain (26–28). In addition to PRC2, the LSD1 complex is another vital partner of HOTAIR, in which lysine-specific demethylase 1(LSD1) is the key subunit (29, 30). The LSD1 complex consists of LSD1, CoREST, and REST, and it can lead to repression of gene expression by reducing the tri-methylation of histone H3 Lys 4(H3K4me3). H3K4me3 is a marker that can target a gene for transcriptional activation, so that H3K4 demethylation is associated with transcriptional inactivation. HOTAIR is capable of binding to the LSD1 complex through a 646 bp fragment in the last exon (**Figure 2**) **(**29). Intriguingly, HOTAIR binds to the PRC2 complex and the LSD1 complex through disparate domains: the 5′ end of HOTAIR (1–300 nt) binds to the RPC2 complex, and the 3′ end of HOTAIR (1,500–2,146 nt) binds to the LSD1 complex. In conclusion, HOTAIR provides a molecular scaffold for the assembly of a gene repressor complex consisting of PRC2 and LSD1, thereby silencing its target gene *via* H3K27 tri-methylation (PRC2 activity) and H3K4 demethylation (LSD1 activity) (17, 29, 31). Apart from functioning as a scaffold for chromatin modifications, HOTAIR also serves as a platform to control protein levels *via* the ubiquitin-proteasome pathway. Specifically, HOTAIR interacts with E3 ubiquitin ligases (Dzip3 and Mex3b) (**Figure 2**) and facilitates the ubiquitination of Ataxin-1 and Snurportin by Dzip3 and Mex3b, respectively, thereby contributing to their degradation (32, 33). Lastly, HOTAIR acts as a competitive endogenous RNA sponge for a wide variety of miRNAs (**Figure 2**) and thereby increases the expression of miRNA-targeted genes (34).

## HOTAIR Functions and Expression in Tumors

**Figure 3:**
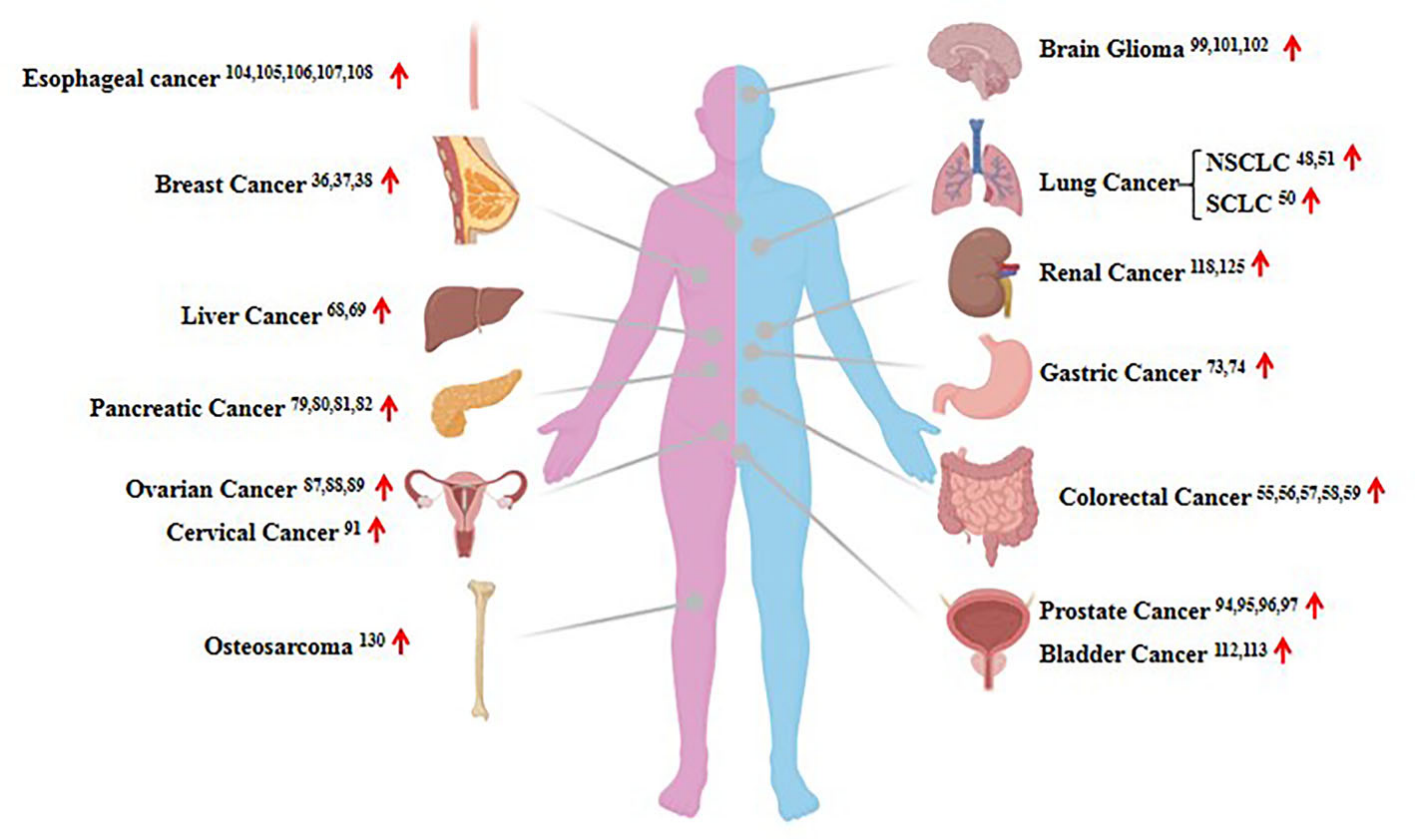
The expression of HOTAIR in human cancers. Shown are examples of the organ-specific expression of HOTAIR. The up red arrow indicates an increase in expression.

Considering HOTAIR can regulate gene expression and protein proteolysis, it has been reported that the lncRNA HOTAIR is dysregulated in the majority of human cancers (**Figure 3**). It has become increasingly obvious that HOTAIR dysregulation in several types of cancer is closely associated with the proliferation, metastasis, and invasion of tumor cells (35, 36). In this review, we describe 14 widely reported human solid tumors associated with HOTAIR dysregulation, and we briefly review the recent data focusing on breast, lung, liver, gastric, and pancreatic cancer and renal cell carcinoma.

### Breast Cancer

Breast cancer (BC) is the most prevalent cancer type in women (37, 38). There were more than two million new cases and over 626,000 deaths worldwide in 2018 (39, 40). HOTAIR expression is overexpressed in various types of breast cancer tissues and cells (41). Arshi (42) reported a significant increase in the level of HOTAIR in BC tissues compared to normal tissues using quantitative reverse transcription polymerase chain reaction (qRT-PCR). Xue et al. (43) reported that HOTAIR was elevated in tamoxifen-resistant BC tissues compared to control tissues. In addition, HOTAIR was also upregulated in serum exosomes of BC patients (44). A recent study revealed that higher HOTAIR was positively associated with BC malignancy but was negatively associated with the radiosensitivity of BC cells (45). These studies implicate the involvement of HOTAIR in the tumorigenesis and progression of BC.

### Lung Cancer

Lung cancer (LC), a malignant tumor, is a serious public health concern worldwide, responsible for more than one million deaths every year (46). Non-small-cell LC (NSCLC) accounts for 80% of LC and is the main type of LC (47), while small-cell LC (SCLC) accounts for ~15% of the remaining cases. Accumulating evidence has suggested that HOTAIR plays a vital role in tumorigenesis, invasion, and metastasis of LC. Zheng et al. (48) reported that HOTAIR was markedly upregulated in NSCLC cells, and elevated HOTAIR promoted migration and invasion of NSCLC cells by increasing epithelial-mesenchymal transition (EMT). Similarly, Ono et al. (49) found that HOTAIR could mediate the invasive phenotype of SCLC cells by promoting EMT, through which HOTAIR could repress the expression of cell adhesion–related genes. These observations showed that higher HOTAIR expression could promote tumorigenesis, metastasis, and invasion of LCs by increasing EMT.

### Liver Cancer

Hepatocellular carcinoma (HCC) is the fifth most prevalent human malignant cancer (50, 51), and the survival rate of HCC patients is still low, with only 30–40% achieving a 1-year survival after surgery (52). Gao et al. (53) reported that HOTAIR was upregulated in HCC tissues compared with adjacent non-cancerous tissues and that elevated HOTAIR contributed to poor tumor differentiation, metastasis, and early recurrence of HCC. Yang et al. (54) also found that the level of HOTAIR was significantly increased in HCC tissues and cell lines, including Hep3B, Huh7, HepG2, and MHCC97H, and elevated HOTAIR promoted migration and invasion of liver cancer cells by enhancing EMT. More importantly, Yang et al. (55) demonstrated that HOTAIR promoted exosome secretion from HCC cells, which subsequently influenced the microenvironment and resulted in tumor progression. Taken together, these observations suggested that HOTAIR could exert oncogenic activity to accelerate the progression of HCC through EMT and the microenvironment.

### Gastric Cancer

Gastric cancer (GC), the second leading cause of cancer-related mortality, is one of the most prevalent cancers worldwide (56). Xiao et al. (57) showed that high levels of HOTAIR could promote proliferation and migration of GC cells through the miR-126/CXCR4 axis and downstream signaling pathways. Furthermore, high HOTAIR expression was closely associated with larger tumor size, extensive metastasis, and advanced pathological stages, and also correlated with shorter overall survival of GC patients. In addition, evidence showed that the suppression of HOTAIR decreased the invasion ability of GC cells by reversing EMT (58). Thus, apart from EMT, HOTAIR could also regulate the proliferation and migration of GC cells through miRNA-mediated signaling pathways.

### Pancreatic Cancer

Pancreatic cancer (PCa) is one of the deadliest cancers. Given the concealed location, it is difficult to detect in its early stages, and the majority of PCa patients are diagnosed at advanced stages, which leads to poor prognosis and high mortality rates. HOTAIR was upregulated in PCa cells and promoted tumorigenesis through epigenetic regulation (29, 59, 60). HOTAIR is overexpressed in both PCa tissues and in cell lines (61). Kim et al. (62) found that HOTAIR was significantly increased in PCa tissues compared with non-tumor tissue, and higher levels of HOTAIR were associated with more aggressive tumors, supporting a pro-oncogenic function of HOTAIR in Pca. Further, elevated HOTAIR increased lactate production, glucose uptake, and ATP production and led to the promotion of PCa cell proliferation (63). Kim et al. (62) demonstrated that high HOTAIR expression could increase PCa cell invasion and proliferation. These observations provide us with new insight indicating HOTAIR increases glucose metabolism and promotes PCa cell proliferation.

### Renal Cell Carcinoma

Renal cell carcinoma (RCC) is a common cancer type, which causes ~90,000 deaths worldwide annually (64). HOTAIR is significantly overexpressed in RCC cell lines and clinical tissues compared with normal cell lines and tissues, and HOTAIR has been associated with tumor progression and clinicopathological characteristics of patients (65). Higher HOTAIR expression promoted proliferation, migration, and invasion of RCC cell lines (66). Moreover, HOTAIR plays an essential role in RCC metastasis. Katayama et al. (67) found that HOTAIR could enhance RCC cell migration by regulating insulin growth factor-binding protein 2 (IGFBP2) expression, and HOTAIR was strongly associated with nuclear grade, lymph-node metastasis, and lung metastasis in RCC. Numerous studies have indicated that HOTAIR could promote RCC malignancy *via* different mechanisms (66, 68–70). More recently, a study (71) indicated that HOTAIR and androgen receptor synergistically promoted tumor angiogenesis and cancer stemness in RCC cells both *in vitro* and *in vivo*.

### Other Solid Cancers

In addition to the six tumors described above, HOTAIR is consistently overexpressed in several other types of cancer, including colorectal cancer (72–75), ovarian cancer (76, 77), cervical cancer (78), prostate cancer (79, 80), brain glioma (81, 82), esophageal cancer (83–85), bladder cancer (86), and osteosarcoma (87–89). Elevated HOTAIR expression can be detected in both cancer cells and tumor tissues through qRT-PCR, *in situ* hybridization, and RNA-sequencing. For example, Tatangelo et al. (90) found that HOTAIR was upregulated in proximal colon cancers by *in situ* hybridization. Chang et al. (79) showed that HOTAIR was overexpressed in the neuroendocrine differentiation (NED) prostate cancer cells and in castration-resistant prostate cancer through RNA-sequencing. Moreover, numerous independent studies have unanimously reported HOTAIR was closely associated with tumorigenesis, tumor staging, metastasis, invasion, proliferation, and apoptosis in human solid cancers. In osteosarcoma, Wang et al. (89) found that HOTAIR was significantly associated with worse histological grade and advanced tumor stage. In the brain glioma, Yang et al. (81) verified that the knockdown of HOTAIR inhibited cell proliferation, promoted cell apoptosis, and suppressed cell invasion and migration during the development of brain glioma. Similarly, in bladder cancer, Yu et al. (86) found that the suppression of HOTAIR inhibited bladder cancer cell proliferation, invasion, and migration, but increased cell apoptosis. Mechanically, HOTAIR played an oncogene function to accelerate tumor progression mainly through epigenetic regulation, EMT, miRNA-mediated downstream signaling pathways, and regulation of other signaling pathways such as the Wnt/β-catenin signaling pathway (83). In addition, HOTAIR may be a reasonable biomarker for predicting tumor risk, diagnosis, and metastasis. Zhang et al. (80) clarified that the level of HOTAIR was significantly higher in bone metastasis tissues than that in the primary prostate cancer tissues, suggesting that HOTAIR could represent a reasonable biomarker for prostate cancer bone metastasis. Further, studies have identified tag single nucleotide polymorphisms (tagSNPs) in HOTAIR and demonstrated that the tagSNPs in HOTAIR were associated with bladder cancer risk in a Chinese population (91). In addition, HOTAIR could serve as a urinary liquid biopsy biomarkers to distinguish bladder cancer from chronic urocystitis (92).

## HOTAIR Prognostic Potential

The correlation between HOTAIR expression and human cancers has been widely reported; however, there is lack of systematic reviews of the prognostic potential of HOTAIR in cancer. Thus, below we summarized recent data relative to the prognostic potential of HOTAIR in terms of drug resistance and survival.

### Drug Resistance

Drug resistance is a major limiting factor in achieving a cure for cancer patients (93), and it also can serve as an indicator to evaluate prognosis. Drug resistance can be roughly divided into treatment drug resistance and chemoradiotherapy resistance. HOTAIR is closely related to the occurrence of drug resistance in several tumors. Fang et al. (94) reported that HOTAIR was overexpressed in SCLC multidrug resistance cells, and HOTAIR knockdown could increase cell sensitivity to anticancer drugs and inhibit tumor growth. HOTAIR was also involved in drug resistance of RCC. Li et al. (95) found that HOTAIR was highly upregulated in sunitinib-resistant cells compared with that in corresponding control cells, and HOTAIR could enhance sunitinib resistance in RCC cells by activating Beclin1-mediated cell autophagy. Moreover, the level of HOTAIR continually increased in prostate cancer cell lines upon treatment with enzalutamide (96), suggesting HOTAIR might serve as a biomarker indicating resistance against enzalutamide.

In addition, overexpressed HOTAIR reduced the sensitivity of tumor cells to chemoradiotherapy. HOTAIR was overexpressed in cisplatin (DDP)-resistant osteosarcoma cells and tissues and enhanced DDP resistance of osteosarcoma cells through the miR-106a-5p/STAT3 axis (97). HOTAIR was also upregulated in the serum exosomes of BC patients and was associated with poor prognosis and poor response to chemotherapy (44). Li et al. (98) demonstrated that high HOTAIR expression could contribute to fluorouracil (5-FU) resistance and was associated with poor response to 5FU treatment in colorectal cancer cells. Özeş et al. (99) revealed that high levels of HOTAIR induced platinum resistance in ovarian cancer. Moreover, the depletion of HOTAIR in HCC cell lines reduced cell susceptibility to TNF-α-induced apoptosis, while it increased the chemotherapeutic sensitivity of liver cancer cells to cisplatin and doxorubicin (100). Additionally, HOTAIR played a critical role in regulating the radiotherapy resistance of tumors. HOTAIR knockdown could increase radiosensitivity of PCa (101) and cervical cancer (102) by regulating autophagy and EMT. Similarly, Liu et al. (103) reported that HOTAIR knockdown potentiated radiosensitivity by regulating the miR-93/ATG12 axis in colorectal cancer.

Taken together, the level of HOTAIR is higher in drug-resistant cancer cells and tissues. Overexpressed HOTAIR could promote the occurrence of drug resistance in cancers and reduce the sensitivity of cancer cells to chemoradiotherapy, indicating HOTAIR may be a vital prognostic factor for predicting drug resistance of tumors.

### Survival

Recently, the level of HOTAIR has been found to be associated with survival in several types of cancer. HOTAIR is closely associated with overall survival, disease-free survival, and survival rates of cancer patients. Martínez-Fernández et al. (104) reported that HOTAIR had prognostic value for bladder cancer progression, recurrence, and survival. Further, the aberrant expression of HOTAIR was associated with poor disease-free survival of bladder cancer (105). Lu et al. (106) measured circulating HOTAIR levels in the serum of 112 BC patients using RT-qRCR and found that BC patients with high circulating HOTAIR showed less clinical response and worse disease-free survival than those with low circulating HOTAIR. In addition, Kim et al. (107) first reported the association between *HOTAIR* gene polymorphisms and colorectal cancer mortality. They chose four *HOTAIR* polymorphisms (rs7958904G>C, rs920778T>C, rs4759314A>G, and rs1899663G>T), conducting genotype frequencies and Cox-regression analysis, and demonstrated that *HOTAIR* rs7958904G>C could be a potent prognostic biomarker for CRC, which was positively associated with CRC prevalence and mortality. These studies suggested overexpressed HOTAIR could decrease disease-free survival and survival rate in some tumors. However, HOTAIR might have the opposite effect. Wang et al. (108) found that HOTAIR was significantly downregulated in patients with primary and acquired resistance to EGFR-TKIs, and in clinical phenotype, they found that high HOTAIR expression was significantly associated with longer progression-free survival compared to low HOTAIR expression subgroup. Nevertheless, the opposite role whereby low HOTAIR expression is associated with unfavorable prognosis in EGFR-TKIs-resistance NSCLCs remains unclear and requires further study.

These studies have indicated that HOTAIR may be used to predict tumor survival, but HOTAIR has diverse effects in different cancers, which needs to be further clarified. The mechanisms underlying HOTAIR tumor survival still need to be further studied.

## Therapeutic Potential of HOTAIR

Numerous studies have emphasized the impact of HOTAIR on tumorigenesis, progression, metastasis, and prognosis of various tumors. Therefore, many therapeutic strategies have been proposed for targeting HOTAIR including silencing HOTAIR expression or function. In terms of HOTAIR silencing, Kim et al. (109) showed that both antisense oligonucleotides (ASOs) and RNA interference (RNAi) could effectively suppress HOTAIR. Gupta et al. (59) used RNAi technology to target HOTAIR, which led to HOTAIR knockout. The loss of HOTAIR could inhibit cancer invasiveness. Besides, Bhan et al. (110) designed a synthetic oligonucleotide DNA as small interfering sense (aiSENSE) that is complementary to the HOTAIR transcript to reduce HOTAIR expression in breast cancer cells. Their results showed that siSENSE could knock down specifically and effectively HOTAIR transcript in breast cancer cells. Apart from directly targeting HOTAIR to reduce its expression, there are some inhibitors that inhibit HOTAIR function without changing HOTAIR level. For example, Li et al. (111) identified a small-molecule compound AC1Q3QWB (AQB) that could disrupt the interaction of HOTAIR-EZH2, and they verified that AQB could selectively and efficiently block PRC2 recruitment. Moreover, Jin et al. (112) studied a novel combination of AQB and CDK4/6 inhibitor palbociclib to evaluate its antitumor effects in glioblastoma. They found that the combination of AQB and Palbociclib had a stronger inhibitory effect on glioma cell growth and metastasis than that in the single drug. Similarly, peptide nucleic acid-PNA3 could disrupt the interaction between HOTAIR and EZH2. Wang et al. (113) disrupted HOTAIR-EZH2 with PNA3 in combination with DNMTi and found that the tumor initiation and stem cell frequency of ovarian cancer stem cells were inhibited, suggesting that dual inhibition of HOTAIR-EZH2 interaction and DNA methylation may be a potent strategy to eradicate ovarian cancer stem cells.

Taken together, the strategies for targeting HOTAIR are mainly through antisense oligonucleotides, RNAi, and small molecule inhibitor, in which antisense oligonucleotides and RNAi directly inhibit HOTAIR expression, while small-molecule inhibitor could block the HOTAIR function. Due to the important role of HOTAIR in tumors, several studies have reported that HOTAIR knockdown may be a potent approach for cancer treatment. HOTAIR knockdown could inhibit DDP resistance of GC cells through blocking the Wnt/β-catenin and PI3K/AKT signaling pathways by upregulating miR-34a (114). Guo et al. (102) reported that HOTAIR knockdown enhanced cervical cancer cell sensitivity to radiotherapy by autophagy reduction and reversal of EMT through inhibiting the Wnt signaling pathway. Similarly, Liu et al. (103) found that HOTAIR knockdown potentiated radiosensitivity of colorectal cancer through regulating the mi-93/ATG12 axis. Moreover, Jia et al. (115) revealed that the HOTAIR/miR-17-5p/PTEN axis might serve as the potential therapeutic strategy for GC. Thus, targeting lncRNA HOTAIR that lead to the suppression of HOTAIR expression or function could serve as a promising therapeutic strategy for several tumors.

## Discussion and Future Perspectives

In recent years, there has been significant progress in clarifying the role of HOTAIR in various physiological and pathological processes. Increasing evidence has suggested that the HOTAIR is overexpressed in a variety of cancers and serves as a potent prognostic factor and therapeutic target in various cancers (**Table 1**). The level of HOTAIR is closely associated with tumor stage, proliferation, migration, and invasion in several human cancers. Moreover, HOTAIR has been demonstrated to affect the drug treatment response and correlates with drug resistance, including chemoradiotherapy resistance. Besides, overexpressed HOTAIR significantly decreased survival of patients in several tumors. These observations suggest that HOTAIR may serve as a potent prognostic factor to predict treatment response and survival rate.

**Table 1:** HOTAIR expression, functions, and prognostic and therapeutic potential in different human cancers.

Cancer type	Expression	Functions	Prognostic and therapeutic potential	Ref.
Breast cancer	Up	Promotes cell growth, invasion, and metastasis.	Poor prognosis, decreases cell radiosensitivity.	(41–45, 106, 116)
Bladder cancer	Up	Promotes proliferation, correlates with invasion.	Poor prognosis, poor DFS.	(86, 91, 92, 104, 105)
Brain glioma	Up	Increases proliferation, invasion, migration, and TNM stage; inhibits apoptosis.	Poor prognosis	(81, 82, 117, 118)
Cervical cancer	Up	Increases proliferation, invasion, metastasis.	Poor prognosis, increases radioresistance.	(78, 102, 119)
Colorectal cancer	Up	Promotes proliferation, metastasis, TNM stage.	Poor prognosis, increases chemo-radioresistance.	(72–75, 90, 98, 103, 107, 120, 121)
Esophageal cancer	Up	Correlates with cell proliferation, advanced stage, invasion.	Poor prognosis, poor OS.	(83, 85, 122–126)
Gastric cancer	Up	Promotes proliferation, invasion, metastasis, TNM stage.	Poor prognosis, increases chemoresistance.	(57, 58, 114, 115, 127, 128)
Liver cancer	Up	Increases proliferation, invasion, migration, EMT, poor differentiation, and exosome secretion.	Poor prognosis, increase chemoresistance.	(53–55, 100)
Lung cancer	Up	Increases proliferation, invasion, and migration and inhibits apoptosis.	Poor prognosis, increases multidrug resistance.	(48, 49, 94, 108, 129–131)
Osteosarcoma	Up	Increases cell growth, invasion, migration; inhibits apoptosis; correlates with advanced stage.	Poor prognosis, increases DDP resistance.	(87–89, 97)
Ovarian cancer	Up	Promotes proliferation, cell cycle, migration, invasion.	Poor prognosis, increases DDP resistance, decreases chemosensitivity.	(76, 77, 99, 132)
Pancreatic cancer	Up	Increases proliferation, invasion, drug resistance; inhibits apoptosis.	Poor prognosis, increases drug resistance, decreases radiosensitivity.	(59, 60, 62, 63, 101, 133, 134)
Prostate cancer	Up	Increases proliferation, invasion, metastasis, and anti-apoptosis.	Poor prognosis, increases drug resistance.	(79, 80, 96, 135)
Renal carcinoma	Up	Increases proliferation, invasion, metastasis, tumor angiogenesis; correlates with TNM stage.	Poor prognosis, increases drug resistance.	(65–67, 69–71, 95, 136)

Based on the close association between HOTAIR and tumorigenesis, progression, and prognosis, targeting HOTAIR may serve as a novel strategy for cancer treatment. Until now, there have been three methods to target HOTAIR—synthetic antisense oligonucleotides, RNAi, and molecule inhibitors that block the interaction between HOTAIR and its partner. In particular, HOTAIR inhibitors mainly focus on blocking the interaction between HOTAIR-EZH2 at present. Thus, more HOTAIR inhibitors still need to be further studied. It has been reported that the HOTAIR knockdown can significantly slow the progression of several tumors and increase their sensitivity to drugs, while additional studies, especially preclinical studies, are needed to prove the therapeutic potential of HOTAIR.

In conclusion, HOTAIR plays a vital role in tumor crucial process such as occurrence, growth, invasion, metastasis, and drug resistance. For this reason, HOTAIR has been regarded as a potential new target for cancer prognosis and therapy. However, the understanding of HOTAIR’s clinical application still needs to be further evaluated to clarify the exact molecular mechanisms underlying dysregulation of its expression and function in different human cancers to provide novel molecules to repress HOTAIR activity in cancer cells. In addition, some studies have shown that HOTAIR can be used in conjunction with currently available drugs to sensitize tumors to the existing therapies, so finding an effective method to target HOTAIR and an efficient drug delivery method *in vivo* would be another critical point. Finally, with more and more studies emerging, lncRNAs such as HOTAIR will act as viable prognostic factors and therapeutic targets for treating human cancers shortly.

## Author Contributions

All authors contributed to the article and approved the submitted version.

## Funding

This work was supported by the Natural Science Foundation of Zhejiang Province [LQ21C060003], the Jin Hua Science and Technology Plan Project [2021-3-148] and the Doctoral Scientific Research Foundation of Zhejiang Normal University [YS304320122].

## Conflict of Interest

The authors declare that the research was conducted in the absence of any commercial or financial relationships that could be construed as a potential conflict of interest.

## Abbreviations

LncRNA, Long non-coding RNA; HOTAIR, HOX transcript antisense RNA; RNA pol II, RNA polymerase II; miRNA, microRNA; PRC2, Polycomb repressive complex 2; LSD1, Lysine-specific demethylase 1; BC, Breast cancer; NSCLC, Non-small-cell lung cancer; SCLC, Small-cell lung cancer; EMT, Epithelial-mesenchymal transition; HCC, Hepatocellular carcinoma; GC, Gastric cancer; PCa, Pancreatic cancer; RCC, Renal cell carcinoma; IGFBP2, Insulin growth factor-binding protein 2; NED, Neuroendocrine differentiation; DDP, Cisplatin; 5-FU, Fluorouracil; ASOs, Antisense oligonucleotides; RNAi, RNA interference; DFS, Disease-free survival; OS, Overall survival.
